# Alterations in Genes of the EGFR Signaling Pathway and Their Relationship to EGFR Tyrosine Kinase Inhibitor Sensitivity in Lung Cancer Cell Lines

**DOI:** 10.1371/journal.pone.0004576

**Published:** 2009-02-24

**Authors:** Jeet Gandhi, Jianling Zhang, Yang Xie, Junichi Soh, Hisayuki Shigematsu, Wei Zhang, Hiromasa Yamamoto, Michael Peyton, Luc Girard, William W. Lockwood, Wan L. Lam, Marileila Varella-Garcia, John D. Minna, Adi F. Gazdar

**Affiliations:** 1 Hamon Center for Therapeutic Oncology Research, University of Texas Southwestern Medical Center at Dallas, Dallas, Texas, United States of America; 2 Department of Pathology, University of Texas Southwestern Medical Center at Dallas, Dallas, Texas, United States of America; 3 Department of Internal Medicine, University of Texas Southwestern Medical Center at Dallas, Dallas, Texas, United States of America; 4 Department of Pharmacology, University of Texas Southwestern Medical Center at Dallas, Dallas, Texas, United States of America; 5 Department of Clinical Sciences, University of Texas Southwestern Medical Center at Dallas, Dallas, Texas, United States of America; 6 Department of Cancer Genetics and Developmental Biology, British Columbia Cancer Research Centre, Vancouver, British Columbia, Canada; 7 Department of Internal Medicine, University of Colorado Cancer Center, Aurora, Colorado, United States of America; University of Florida, United States of America

## Abstract

**Background:**

Deregulation of *EGFR* signaling is common in non-small cell lung cancers (NSCLC) and this finding led to the development of tyrosine kinase inhibitors (TKIs) that are highly effective in a subset of NSCLC. Mutations of *EGFR* (m*EGFR*) and copy number gains (CNGs) of *EGFR* (g*EGFR*) and *HER2* (g*HER2*) have been reported to predict for TKI response. Mutations in *KRAS* (m*KRAS*) are associated with primary resistance to TKIs.

**Methodology/Principal Findings:**

We investigated the relationship between mutations, CNGs and response to TKIs in a large panel of NSCLC cell lines. Genes studied were *EGFR*, *HER2*, *HER3 HER4*, *KRAS*, *BRAF* and *PIK3CA*. Mutations were detected by sequencing, while CNGs were determined by quantitative PCR (qPCR), fluorescence in situ hybridization (FISH) and array comparative genomic hybridization (aCGH). IC50 values for the TKIs gefitinib (Iressa) and erlotinib (Tarceva) were determined by MTS assay. For any of the seven genes tested, mutations (39/77, 50.6%), copy number gains (50/77, 64.9%) or either (65/77, 84.4%) were frequent in NSCLC lines. Mutations of *EGFR* (13%) and *KRAS* (24.7%) were frequent, while they were less frequent for the other genes. The three techniques for determining CNG were well correlated, and qPCR data were used for further analyses. CNGs were relatively frequent for *EGFR* and *KRAS* in adenocarcinomas. While mutations were largely mutually exclusive, CNGs were not. *EGFR* and *KRAS* mutant lines frequently demonstrated mutant allele specific imbalance i.e. the mutant form was usually in great excess compared to the wild type form. On a molar basis, sensitivity to gefitinib and erlotinib were highly correlated. Multivariate analyses led to the following results:

1. m*EGFR* and g*EGFR* and g*HER2* were independent factors related to gefitinib sensitivity, in descending order of importance.

2. m*KRAS* was associated with increased in vitro resistance to gefitinib.

**Conclusions/Significance:**

Our in vitro studies confirm and extend clinical observations and demonstrate the relative importance of both *EGFR* mutations and CNGs and *HER2* CNGs in the sensitivity to TKIs.

## Introduction

Lung cancer is the leading cause of all cancer deaths worldwide [1]. Despite the recent advances in diagnosis and multimodality therapies for lung cancers, the prognosis still remains poor with 5-year survival rates of only 16% for all stages [2].

Lung cancer is characterized by the accumulation of multiple genetic and epigenetic alterations including somatic mutations and gene copy number gains or both which results in the activation of oncogenes or inactivation of tumor suppressor genes [3]. Epidermal growth factor receptor (*EGFR*) deregulation has been observed in multiple tumor types including non-small cell lung cancers (NSCLCs) [4]. Hirsch et. al. identified frequent *EGFR* protein over expression (62%) in NSCLCs of squamous cell and adenocarcinoma subtypes [5]. *EGFR* over expression is often associated with adverse prognosis [6]. The receptor tyrosine kinase (RTK) super-family of cell surface receptors serves as mediators of cell signaling by extra-cellular growth factors. Members of the ERBB family of RTKs including *EGFR(HER1/ERBB1)*, *HER2 (ERBB2/EGFR2)*, *HER3 (ERBB3/EGFR3)* and *HER4 (ERBB4/EGFR4)* have received much attention given their strong association with malignant proliferation.

The RAS/MAPK and PI3K/AKT pathways are major signaling networks linking *EGFR* activation to cell proliferation and survival [7]. As discussed below, *EGFR* signaling pathway genes have been reported to be mutated in NSCLC. Depending on the geographical location, *EGFR* and *KRAS* mutations have been identified in ∼10% −30% of NSCLCs [3], [8]. *EGFR* mutations are independently associated with adenocarcinoma histology, East Asian ethnicity, never smoking status and female gender. Mutations of *KRAS* also target adenocarcinoma histology, but otherwise differ from *EGFR* mutations because they are relatively rare in East Asians and occur more frequently in males and smokers [9]. Less commonly, somatic mutations have also been found in other *EGFR* pathway genes including *HER2* (∼2%) [10], *HER4* (∼2%) [11], *BRAF* (∼2%) [12], and *PIK3CA* (∼4%) [13], [14].

Gene copy number gains (CNGs) due to focal amplification or chromosomal polysomy, is one of the other major mechanisms of oncogene activation [15]. Lockwood et al. identified multiple components of the *EGFR* pathway signaling were frequently amplified and over-expressed in NSCLC. Interestingly, they also found that *EGFR* pathway gene amplification was more frequent in the adenocarcinoma subtype of NSCLC.

Because of the frequent deregulation of *EGFR* pathway genes in NSCLC, EGFR became one of the first rationally selected molecules for targeted therapy. While initial targeted approaches utilized monoclonal antibodies which block the ligand-receptor interaction, newer approaches have utilized small molecule reversible tyrosine kinase inhibitors (TKIs). The tyrosine kinase activity of *EGFR* is required for the biochemical responses induced by this receptor [7], [16], [17]. Two TKIs, gefitinib (Iressa, AstraZeneca) and erlotinib (Tarceva, Genentech) have been widely used in the treatment of advanced or recurrent NSCLC. Responses were noted in subsets, notably East Asian ethnicity, female gender, never smoking status and adenocarcinoma histology [18], [19], [20]. Subsequently, *EGFR* mutations were identified in the tyrosine kinase domain, and predicted for response to TKI in the same subset of patients [21], [22], [23]. According to a meta-analysis of 1170 patients, more than 70% of NSCLCs with *EGFR* mutations responded to TKIs, whereas 10% of tumors without *EGFR* mutation responded [24]. However, further studies indicated that factors other than *EGFR* mutations may play a role in determining response and survival after TKI therapy. *EGFR* gene copy number gain was associated with significantly improved TKI sensitivity and survival in a large unselected study with appropriate controls [25]. In addition, other members of the *EGFR* family i.e. *HER2* [26] and *EGFR3* [27] may be important factors involved in TKI sensitivity. A further complexity is the clinical observation that somatic mutations of *KRAS* confer intrinsic resistance to TKIs [28].

To further understand the relationship between TKI sensitivity and deregulation of *EGFR* pathway genes, we examined the mutation and copy number status of seven of these genes (*EGFR*, *HER2*, *HER3*, *HER4*, *KRAS*, *BRAF*, and *PIK3CA*) in a large panel of lung cancer cell lines and correlated the data with in vitro sensitivity to TKIs.

## Materials and Methods

### Tumor Cell Lines

We studied a total of 112 cell lines consisting of 77 NSCLC and 32 small cell lung cancer (SCLC) and three lines from small cell cancers at extrapulmonary sites (extrapulmonary small cell cancers, ExPuSC) [29]. All except three of these cell lines were established by the authors [30] at one of two locations. Cell lines initiated at the NCI have the prefix NCI-H while lines established at UT Southwestern have the prefix HCC. NCI-H3255 was obtained from Dr. Bruce Johnson (Lowe Center for Thoracic Oncology, Dana-Farber Cancer Institute, Boston, MA) [22]. PC-9 (originally from the Tokyo Medical University, Tokyo, Japan) was obtained from Dr. Bert Vogelstein (Johns Hopkins University School of Medicine, Baltimore, MD). Calu-3 was purchased from American Type Culture Center (Manassas, VA). We also investigated eight immortalized human bronchial epithelial cell lines (HBECs, HBEC1KT, HBEC3KT, HBEC4KT, HBEC5KT, HBEC17KT, HBEC30KT, HBEC31KT and HBEC34KT), which were initiated by us [31].

Most of the tumor cell lines were maintained in RPMI1640 supplemented with 5–10% fetal bovine serum (FBS). A few cell lines were grown in ACL4 (for NSCLC lines) and HITES (for SCLC lines) supplemented with 5% FBS. All HBEC cell lines were maintained in Keratinocyte-SFM (Invitrogen, Carlsbad, CA) with bovine pituitary extract (BPE) and recombinant epidermal growth factor (EGF) [31]. All cell lines were incubated at 37°C in a humidified atmosphere with 5% CO_2_.

The genetic fingerprint of each cell line was obtained (Powerplex 1.2 system, Promega, Madison, WI) and each cell line had a unique genetic profile which was identical to the profiles obtained from the American Type Culture Collection.

### DNA and RNA extraction

Genomic DNA was obtained from cell line pellets by standard phenol-chloroform (1∶1) extraction followed by ethanol precipitation [32] or by using DNeasy Tissue Kit (QIAGEN, Valencia, CA). Total RNA was obtained from cell lines using RNeasy Plus Mini Kit (QIAGEN). cDNA was prepared as described previously [14].

### Gene Sequencing

DNA sequencing and mutational analysis for *EGFR* (exons 18–21), *HER2* (exons 19 and 20), *KRAS* (codons 12, 13, and 61), *BRAF* (exons 11 and 15) and *PIK3CA* (exons 9 and 20) were done as reported by us previously [10], [14], [32]. *HER3* (exons 18–21) and *HER4* (exons18–23) mutation status were analyzed by PCR amplification of genomic DNA or cDNA and direct sequencing of the PCR products [10]. The mutations in these genes were determined using the PCR primers as listed (Table S6). All PCRs were performed in 25 µL volumes containing 100 ng of DNA using HotStarTaq DNA polymerase (QIAGEN Inc., Valencia, CA). DNA was amplified for 32 to 34 cycles at 95°C for 30 seconds, 62°C to 68°C for 30 seconds, and 72°C for 30 seconds followed by 7 minutes extension at 72°C. All PCR products were incubated using exonuclease I and shrimp alkaline phosphatase (Amersham Biosciences Co., Piscataway, NJ) and sequenced directly using Applied Biosystems PRISM dye terminator cycle sequencing method (Perkin-Elmer Co., Foster City, CA). All mutations were confirmed by independent sequencing in both directions.

### Copy number evaluation

Copy number gains can be evaluated by a number of methodologies. For clinical samples, fluorescence in situ hybridization (FISH) is frequently used as it can discriminate between tumor and non-malignant cells. Laboratory studies of tumor cell lines (which are free of non-malignant cells) often utilize quantitative PCR (qPCR). An alternative method is array comparative genomic hybridization (aCGH). Because direct comparisons of these methods have seldom been reported [33], we utilized all three methods.

### Real-time qPCR

Gene copy numbers were determined using real-time quantitative PCR employing the Chromo4 PCR System (Bio-Rad Laboratories, Hercules, CA). To determine the copy number of a target gene, we used control genes located on the same chromosome as the target gene (Table S6). The resultant ratios were compared to similar ratios from diploid cells (the mean values of the eight HBEC cell lines. TaqMan methodology was used except for *PIK3CA* [14]. Primers and probes were chosen by TaqMan Primer Express™ 1.5 (Applied Biosystem, Foster City, CA). Primers were purchased from Invitrogen and probes from Biosearch Technologies (Novato, CA). The sequences of primers and probes are shown in Table S6. Standard curves were constructed with serial dilutions of specific PCR products. Amplification mixes (25 µl) contained the sample DNA (20 ng), 10× TaqMan buffer (2.5 µl), 200 µM dNTP, 1.25 U Hotstar Taq™ DNA polymerase, 200 nM each primer and 100 nM probe. The thermal cycling conditions comprised 5 min at 95°C and 40 cycles at 95°C for 15 s and 60°C for 30 s. All the samples were analyzed in triplicate. The parameter C_t_ is defined as the fractional cycle number at which the fluorescence generated by cleavage of the probe passes a fixed threshold above baseline. The target gene copy number in unknown samples is quantified by measuring C_t_ values and by using a standard curve to determine the copy number [34]. Gene copy number was calculated using the following equation: g = (S_T_/S_C_)/(N_T_/N_C_). *PIK3CA* copy number was assessed as described by us previously [14].

### Tiling path aCGH

aCGH was performed as previously described [14], [15]. Genomic imbalances were identified using aCGH-Smooth [35] as previously described [36].

### FISH assays

Dual-color FISH assays were performed according to a standard protocol [37], [38]. The probe sets, EGFR/CEP7 and PathVysion and the controls CEP7 were obtained from Abbott Molecular (Des Plaines, Illinois), HER3/CEP12 was obtained from QBiogene; BRAF probe was generated from the bacterial artificial clone (BAC) clone used for aCGH. The copy numbers of the target genes (labeled in red fluorophores) and the CEP probes (labeled in Spectrum Green) were verified in at least 100 interphase cells and 20 metaphase spreads. Images were captured and merged using the CytoVision workstation (Applied Imaging, San Jose, CA).

### Sub-cloning

Genomic DNA was isolated from mutant *EGFR* or *KRAS* NSCLC cell lines and used as a template for PCR amplification of *EGFR* or *KRAS*. The primers and the conditions of PCR reactions were as described previously. PCR products were cloned into pCR2.1-TOPO vector using TOPO TA cloning kit (Invitrogen). About 20 clones (range15–25) were selected for sequencing using either M13 forward primer or corresponding *EGFR* or *KRAS* primers. The results are expressed as the percentages of mutant alleles present in the total number cloned.

### TKI sensitivity

The MTS colorimetric assay (Promega) was performed as per the manufacturer's instructions. This assay is based on the conversion of MTS into soluble formazan by endogenous dehydrogenase enzymes found in metabolically active cells. Cells were plated at 1×10^3^–4×10^3^ cells/well in tissue culture treated 96-well plates. The following day, TKI (gefitinib or erlotinib) was added to each plate in a dilution series across the plate such that eight different concentrations of the drug were tested. On day 5, 20 µl of MTS was added, followed by a 1 hour incubation at 37°C and then the absorbance was read at 490 nm on a plate reader.

96-well plate data were imported into an in-house Database of In VItro drug Sensitivity Assays (DIVISA by Luc Girard, manuscript in preparation) where IC50s are calculated as well as various statistical analyses. To calculate IC50 values, the data were background-subtracted (columns 1 and 12 typically contained media with no cells or drugs and served as background values), and fitted to the DRC model (R package ‘drc’ by Christian Ritz and Jens Streibig, http://www.bioassay.dk) to generate a sigmoidal curve from which the concentration that inhibits 50% of the cells (IC50) was determined.

### Statistical Analyses

Fisher's two-tailed exact tests were determined using the Prism 4 software (Graph Pad, San Diego, CA). P values<0.05 were considered significant. Other statistical analyses are discussed under appropriate categories in the Results section.

## Results

### Mutations (m) and CNGs (g) in lung cancer cell lines

We examined 32 SCLC and three lines from small cell cancers from extrapulmonary sites (extrapulmonary small cell cancers, ExPuSC), for somatic mutations and CNGs of *EGFR* pathway genes. We found a total of only three mutations in 35 cell lines and all three were *PIK3CA* mutations (Table S1). CNGs for *EGFR* pathway genes were infrequently encountered in SCLC (Table S2). Since somatic mutations and CNGs for *EGFR* pathway genes were rare in SCLC, we limited our further studies to NSCLC.

For any of the seven genes tested, mutations (39/77, 50.6%), copy number gains (50/77, 64.9%) or either (65/77, 84.4%) were frequent in NSCLC lines. These findings are discussed in greater detail below.

### Mutations (m) of *EGFR* pathway genes in NSCLC

**Figure 1:**
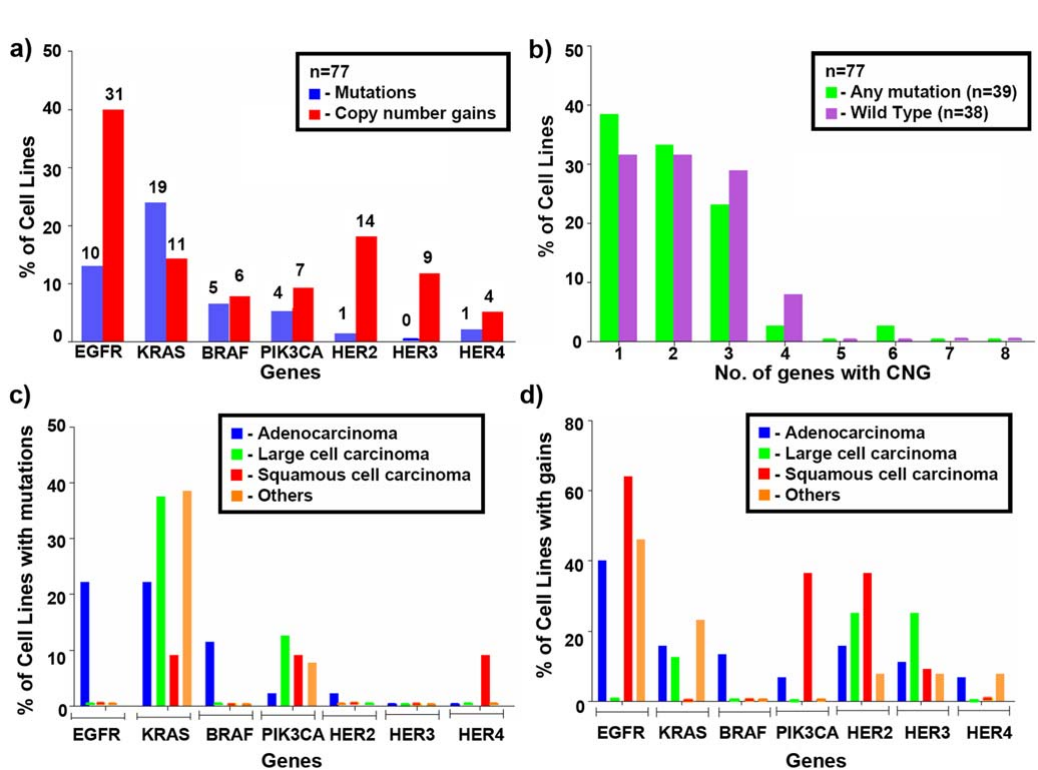
Mutations (m) and Copy number gains (g) of *EGFR* pathway genes in NSCLC. Fig 1a. shows the frequency of mutations and copy number gains of *EGFR* pathway genes (*EGFR*, *KRAS*, *BRAF*, *PIK3CA*, *HER2*, *HER3* and *HER4*). Forty mutations were identified in 39 cell lines. Mutations and copy number gains were more frequent for *EGFR* (13%, 40.3%) and *KRAS* (24.7%, 14.3%) than other gene. CNGs for *HER2* (18.2%) were also common. We identified only one *HER2* and one *HER4* somatic mutation. The numbers above the columns indicate the number of cell lines with mutations (blue columns) or copy number gains (red columns). Fig 1b. The figure depicts the number of genes demonstrating CNGs in mutant and wild type cell lines. Of the 77 cell lines examined, 39 (50.6%) had a mutation in at least one of seven the EGFR pathway genes examined. CNGs were frequent in both mutant and wild type cell lines. Fig 1c. shows frequency of mutations on the basis of NSCLC subtype. Mutations of *EGFR* and *BRAF* were exclusively found in adenocarcinoma subtype. The single *HER2* mutation was in a adenocarcinoma as compared to the *HER4* somatic mutation which was identified in a squamous cell ca. Fig 1d. shows frequency of copy number gains (CNGs) (g>4 by qPCR) on the basis of NSCLC subtype. CNGs for *BRAF* and *PIK3CA* were seen predominantly in adenocarcinoma and squamous cell carcinoma respectively. CNGs for the rest of the genes did not favor any subtype.

We examined the NSCLC lines, for somatic mutations of *EGFR* pathway genes as listed in the Methods section. We found a total of 40 mutations in 39 (50.6%) cell lines (Fig 1a, Table S3). These mutations consisted of 19 *KRAS* (24.7%), 10 *EGFR* (13%), five *BRAF* (6.5%), four *PIK3CA* (5.2%), one *HER2* (1.3%), one *HER4* (2.2%) and none for *HER3*. m*EGFR*, m*BRAF* and m*HER2* were present exclusively in adenocarcinomas while m*KRAS* were more frequent in adenocarcinoma and large cell histologies. m*PIK3CA* were the only mutations that did not target adenocarcinoma histology (Fig. 1c).

### Mutations are mutually exclusive

We examined for any association between somatic mutations in individual cell lines. In order to test the hypothesis that *EGFR* pathway gene mutations are mutually exclusive, we used Monte Carlo simulations to calculate the empirical p-value. The null hypothesis is that mutations will occur independently. We simulated the joint distribution of mutation events among these seven genes using the observed marginal mutation rates and the independent assumption. In each simulation, we noted the number of cell lines with multiple mutations. We repeated the simulations 10,000 times and obtained the empirical distribution of the number of cell lines with multiple mutations; then we compared the observed number of cell lines with multiple mutations with this empirical distribution to calculate the p-value.

For this study, the one-sided p-value is 0.0014; therefore, we rejected the null hypothesis and concluded that gene mutations are mutually exclusive events for these genes in NSCLC cell lines, with one exception (Table 1). Large cell carcinoma line NCI-H460 had both *KRAS* and *PIK3CA* mutations.

**Table 1:** Are mutations of *EGFR* pathway genes mutually exclusive?

	WT	m*EGFR*	m*KRAS*	m*PIK3CA*	m*BRAF*	m*HER2*	m*HER4*
WT	**38**	0	0	0	0	0	0
m*EGFR*	0	**10**	0	0	0	0	0
m*KRAS*	0	0	**18**	**1**	0	0	0
m*PIK3CA*	0	0	**1**	**3**	0	0	0
m*BRAF*	0	0	0	0	**5**	0	0
m*HER2*	0	0	0	0	0	**1**	0
m*HER4*	0	0	0	0	0	0	**1**

Table 1 shows that mutations of *EGFR* pathway genes are mutually exclusive in NSCLC (p<0.05). The only exception was a cell line which harbored mutations for both *KRAS* and *PIK3CA* mutations.

### Comparison of methods for determining copy number gains

aCGH, FISH and qPCR were used to test the gene copy numbers for NSCLC cell lines. There is no clear biological threshold value for defining the abnormal copy numbers for NSCLC human cell lines; we selected the threshold values which could achieve the best positive or negative category agreement among the three tests. Specifically, we computed the Kappa statistics [39] between aCGH and qPCR tests over two dimensional cut-off grids as 2, 2.5, 3, 3.5, 4, 4.5 and 5; and then did the same for FISH and qPCR. The cut-off values that achieved the best agreement among three tests were as follows: 4 for qPCR, 3 for CGH and 4.5 for FISH; and we used these values to define the presence of copy number gains in NSCLC cell lines. Using these cut-off values and all available data, there was highly significant concordance (p<0.001) between the three methods as shown in Table 2.

**Table 2:** Comparison of the three methods used for studying gene copy number.

Comparison	Number of correlations	Concordance (%)	Fisher two-sided p-value
**qPCR vs aCGH**	317	89.3	<0.001
**qPCR vs FISH**	99	71.7	<0.001
**aCGH vs FISH**	72	76.4	<0.001

Gene copy number was measured using three methods, qPCR, aCGH and FISH. All samples used in this study had qPCR data and subset data for the other two methods. All available data were pooled and used for analysis of concordance and kappa statistics. All three comparisons show high concordance with low p values. Kappa analyses were used to determine the optimal cut-off value for each test. These analyses yielded the following cut-off values for determining copy number gains: qPCR≥4.0, aCGH≥3.0 and for FISH≥4.5. Using these cut-off values, samples were scored as positive or negative for copy number gains for each type of test, and Fishers two sided tests were used for comparisons of the different tests.

### *EGFR* pathway gene CNGs

As the qPCR data were complete for all lines (while subsets were tested by the other two methods which are more laborious and expensive), we used qPCR data for all further analyses. Copy number gains were frequent (>10%) for *EGFR*, *HER2*, *HER3* and *KRAS* (Fig 1a, Table S4). In particular gains for *EGFR* were more than twice as frequent (40%) than for any other gene. In general, with the exceptions of g*BRAF* (limited to adenocarcinoma histology), and g*PIK3CA* (largely limited to squamous cell histology), CNGs did not show any apparent histology subtype bias (Fig. 1c).

### Relationship between Mutations and Copy number gains

**Figure 2:**
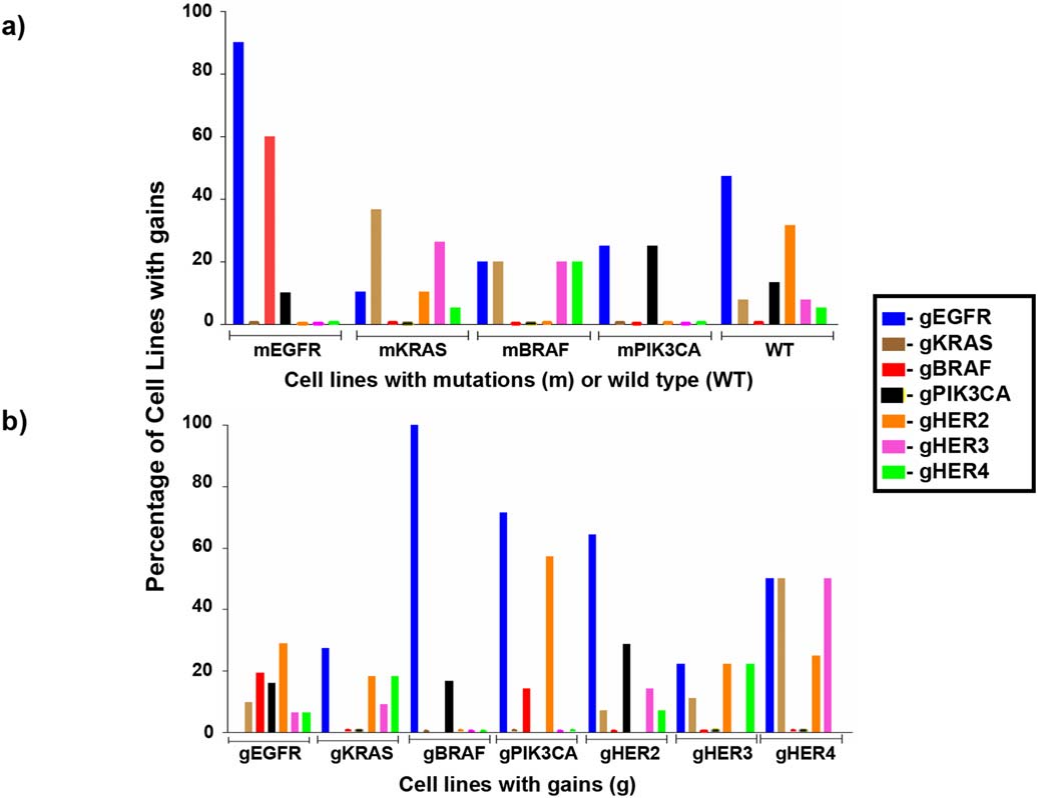
Copy number gains are not mutually exclusive with either other copy number gains or with mutations. Fig 2a. shows that copy number gains and mutations are not mutually exclusive. As evident from the figure CNGs for *EGFR* and *KRAS* are significantly more frequent in *EGFR* and *KRAS* mutant cell lines respectively (p<0.05). There was only one *HER2* and *HER4* mutant NSCLC cell line and thus they were not included in this figure. Fig 2b. shows that copy number gains are not mutually exclusive and gains of one gene may occur in the presence of gains for other genes.

**Figure 3:**
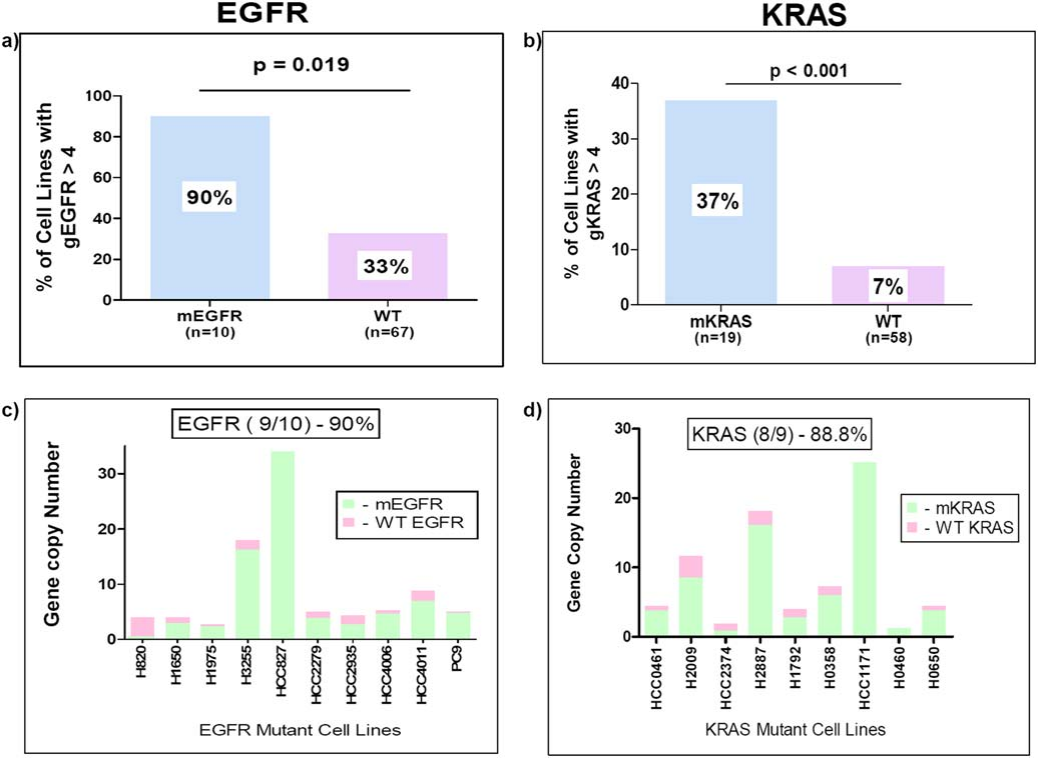
Mutant allele specific imbalance (MASI) for *EGFR* and *KRAS* genes. *EGFR* and *KRAS* genes preferentially have copy number gains (CNG) in cell lines harboring the respective mutations (panels a and b). In mutant lines, the mutant allele almost always is in excess compared to the wild type allele (panels c and d), a phenomenon we have termed MASI. In most MASI cases the mutant allele demonstrates CNGs; however MASI may also be present in cell lines having a diploid copy number of the oncogene, (acquired uniparental disomy) either uniform (NCI-H460) or heterogeneous (NCI-H1975).

Unlike mutations, copy number gains were not mutually exclusive either with other CNGs or with mutations (Fig. 2). We found CNGs for two or more genes in 32.5% (25/77) of cell lines (Table S4). However, we noted a highly significant association between mutations of *EGFR* or *KRAS* and CNGs of their respective genes (Fig 3a, 3b).

### Mutant allele specific imbalance (MASI) for *EGFR* and *KRAS* genes

As noted above CNGs of *EGFR* and *KRAS* were more frequent in cell lines harboring these mutations. Employing the sub-cloning method previously described, we investigated whether the *EGFR* and *KRAS* genes preferentially demonstrated mutant allele specific CNGs in cell lines harboring the respective mutations (Fig. 3). The data, for testing whether mutant alleles are amplified in gene mutant cell lines are clustered data with binary outcome. Each cell line is a cluster and the null hypothesis is that the mutation rate is 0.5. Because the number of subclones in each cell line varied, we used the analysis approach for the clustered binary outcome with various cluster sizes [40]. In mutant lines, the mutant allele was almost always in excess compared to the wild type allele, a phenomenon we have termed MASI. The p-value for *EGFR* mutant cell lines was 0.019 and for *KRAS* mutant cell line was 0.0003 indicating that the mutant alleles were preferentially dominant in both cases. Most MASI cases were due to increased copy number of the mutant allele. However, in a minority of cases MASI was also noted in cells having diploid or near diploid copy numbers (acquired uniparental disomy). Thus we used the term MASI as opposed to mutant allele specific gains.

### Characteristics of *EGFR* Mutant Cell Lines

The clinico-pathologic and molecular data for the 10 NSCLC cell lines which harbor *EGFR* mutations are summarized in Table 3. All contained one of the two major mutations in the kinase domain, either deletions of exon 19 (n = 7) or L858R mutation in exon 21 (n = 3). Mutations were exclusively seen in adenocarcinoma and never smokers or patients with low tobacco exposure (≤10 pack years). One *EGFR* mutant cell line was developed in Japan and the remaining nine were developed in North America. Of the nine developed in North America, only one was from an individual of Asian ethnicity. In addition, we identified that *EGFR* mutations were more common in comparatively younger age group (<55 years).

**Table 3:** Characteristics of *EGFR* Mutant Cell Lines.

Cell Line	Subtype	Sex	Race	Age	Pack Years	Mutation 1		Mutation 2	*EGFR* Copy # (qPCR)	Gefitinib IC50	Rank Order
						Exon	Mutation				
PC-9	AD	?	EA	42	0	19	Del E746-A750	None	5	0.03	1
HCC0827	AD	F	W	40	4	19	Del E746-A750	None	34	0.04	3
HCC2279	AD	F	EA	52	?	19	Del E746-A750	None	5	0.05	4
H3255	AD	F	W	?	?	21	L858R	None	18	0.09	5
HCC2935	AD	M	W	39	0	19	Del E746-S752	None	4.4	0.1	6
HCC4006	AD	M	W	52	0	19	Del E746-A750	None	5.2	0.2	7
HCC4011	AD	M	W	53	5	21	L858R	None	8.8	0.5	8
H820	AD	M	W	53	?	19	Del E746-E749	T790M	4	3.0	10
H1650	AD	M	W	27	10	19	Del E746-A750	PTEN Del −/−	4	11.7	12
H1975	AD	F	?	?	?	21	L858R	T790M	2.8	25.0	26

Table 3 shows the clinico-pathologic and molecular data for the 10 NSCLC cell lines, which harbor *EGFR* mutations. These cell lines are arranged in decreasing order of gefitinib sensitivity. Mutations were exclusively seen in adenocarcinoma and never smokers or patients with low tobacco exposure (≤10 pack years or PY). The primary activating *EGFR* mutations in all 10 cases were either deletions of exon 19 (n = 7) or L858R mutation in exon 21 (n = 3). Three cell lines also had a second mutation, either T790M resistance associated mutation in exon 20 (n = 2) or homozygous deletion of the *PTEN* gene. Contradictory data are available for the gender for the patient from whom the PC-9 cell line was originated – the surgeon informed us that the patient was female while the distributing institution states that it was from a male (personal communication from Dr. Harubumi Kato, Tokyo Medical University, Japan). Cell lines considered resistant to gefitinib (IC50>1 µM) are indicated in red. Rank order is the sensitivity of cell lines to gefitinib in descending order with 1 being the most sensitive. Abbreviations: EA – East Asian, W – White, PY – pack years, Del – deletion.

Seven of these cell lines were sensitive to gefitinib. The three resistant lines had a second mutation: either the T790M resistance associated mutation in exon 20 (n = 2) or a homozygous deletion of the *PTEN* gene and absence of its protein [41] (authors' unpublished observations).

### Effects of mutations and copy number gains on sensitivity to TKIs

To evaluate the effect of mutations and copy number gains on sensitivity to TKIs, we analyzed the IC50 values of 45 NSCLC cell lines. Because TKI clinical sensitivity preferentially targets adenocarcinoma histology, we included 31 adenocarcinomas. The entire subset included all of the *EGFR*, *HER2* and *HER4* mutant cell lines and 12 *KRAS* and 3 *BRAF* cell lines. Because *PIK3CA* mutations favored non-adenocarcinoma histology only one *PIK3CA* mutant cell line was included. Of the entire subset 17 lines were wild type for all genes tested (Table S5).

**Figure 4:**
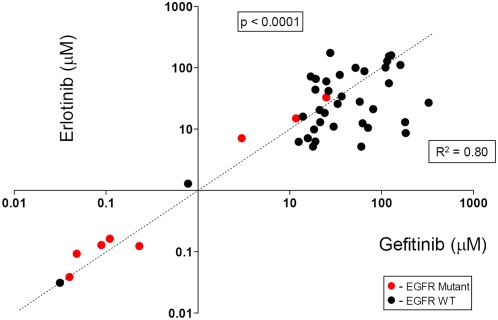
Concordance between IC50 values for gefitinib vs erlotinib. Forty five cell lines were tested for sensitivity to both drugs and the concordance was excellent (p<0.0001).

We found an excellent concordance between the IC50 values between gefitinib and erlotinib (p<0.0001) (Fig 4, Table S7). Because our data set for gefitinib sensitivity was more extensive we elected to utilize the gefitinib data for further analyses.

**Figure 5:**
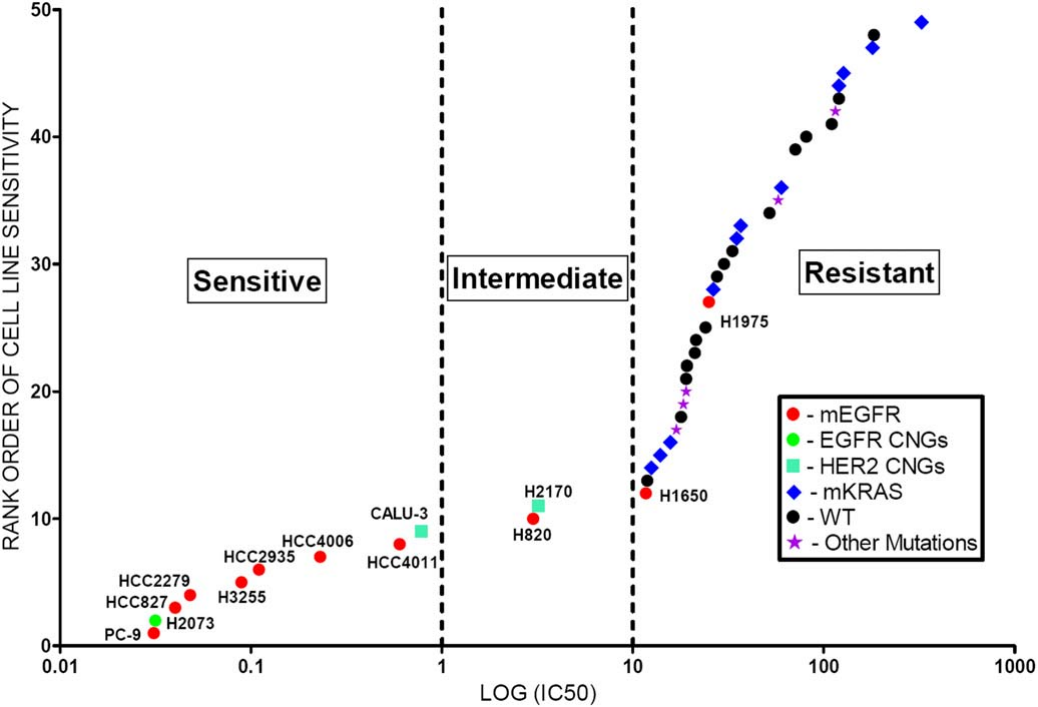
Rank Order of NSCLC Cell Line depending on the Iressa IC50. Fig 5. shows a log curve of the gefitinib IC50 values for 45 NSCLC cell lines. They are classified into three categories on the basis of gefitinib IC50: Sensitive (IC50<1 µM), Intermediate (IC50>1 but <10 µM) and Resistant (IC50>10 µM). Of the nine sensitive cell lines, seven of them harbor *EGFR* mutations, one has CNGs for *EGFR* and one has CNG for *HER2*. Of the remaining *EGFR* mutant cell lines, two had T790M mutation (one intermediate and one resistant) and one had a homozygous deletion of *PTEN* (resistant). *KRAS* mutant and wild type cell lines were all resistant to gefitinib.

Fig. 5 illustrates the sensitivity patterns of the tested cell lines. The in vitro concentration of 1 µM used in tissue culture correlates to the plasma concentration of gefitinib in patients treated with the standard dose of gefitinib i.e. 250 mg a day. This threshold has been used by researchers in the past to distinguish sensitive from insensitive and/or resistant cell lines [7]. On the basis of the above-mentioned threshold and the shape of the curve, we divided our cell lines into 3 categories: sensitive (≤1 µM), intermediate (>1 µM but ≤10 µM) and resistant (>10 µM) as seen in figure Fig. 5. The IC50 values follow a line that demonstrated a slope change point at approximately 10 µM, and thus demonstrated an apparently bimodal distribution (Fig. 5). As cell lines with values below 1 µM were regarded as sensitive, we arbitrarily categorized values between 1 and 10 µM as being intermediate in sensitivity.

There were nine cell lines in the sensitive group and they included seven of the ten m*EGFR* lines, one g*EGFR* cell line and one g*HER2* cell line. The intermediate category consisted of only two cell lines; a m*EGFR* cell line having a secondary T790M mutation and a g*HER2* cell line. The resistant cell line category was the largest and included two m*EGFR* cell lines, one having the secondary T790M mutation and the other having homozygous deletion of *PTEN* gene. The remaining resistant lines included all of the wild type lines and all lines having *KRAS*, *BRAF*, *HER2*, *HER4* and *PIK3CA* mutations.

Using univariate tests, we analyzed the association between gefitinib sensitivity and mutational status or CNGs of the various *EGFR* pathway genes and found a significant correlation between gefitinib sensitivity with *EGFR* mutation (p = 0.002) and *EGFR* copy number gains (p = 0.001). We tested the hypothesis that KRAS mutations confer intrinsic resistance to TKIs. Because m*EGFR* was associated with sensitivity to TKIs we excluded these lines from our analyses. We compared the rank order of IC50s of m*KRAS* cell lines with a) all other cell lines and b) cell lines wild type for all other tested genes. Our univariate analyses demonstrated that indeed *KRAS* mutations conferred in vitro resistance to gefitinib (Table 5). None of the other gene mutations or copy number gains showed any significant correlation with gefitinib sensitivity.

We then used a multivariate linear regression model [42] to explore the association between gefitinib sensitivity and *EGFR* mutation, *EGFR* copy number and *HER2* copy number (Table 4). The response variable was log IC50 values of gefitinib (as a continuous variable), the predictors are *EGFR* mutation status (mutation or wild type *EGFR*), *EGFR* copy number (measured by qPCR) and *HER2* copy number (measured by qPCR) as continuous variables. After adjusting for the effects of *EGFR* and *HER2* copy numbers, the *EGFR* mutation status was significantly associated with the gefitinib sensitivity (p<0.001). Similarly after adjusting for the effects of the other two variables, the *EGFR* copy number (p value = 0.002) and *HER2* copy number (p = 0.021) were independently and significantly associated with the gefitinib sensitivity. To summarize the multivariate analysis, all 3 parameters, i.e. m*EGFR* mutations, g*EGFR* and g*HER2*, showed a correlation with TKI sensitivity in decreasing order of importance. We also repeated the analysis using the copy number of *EGFR* and *HER2* as binary variables. In these analyses, *EGFR* copy number remained significantly associated with gefitinib sensitivity (p value<0.001) while *HER2* copy number was no longer significantly associated (p value>0.05; Table 4).

**Table 4:** Results of Multivariate Regression Analyses.

Variable	Estimate*	Standard Error	p-value
***EGFR*** ** Mutation**	−1.50	0.28	<.0001
***EGFR*** ** relative copy number**	−0.07	0.02	0.002
***HER2*** ** relative copy number**	−0.01	0.004	0.021

From the multivariate regression analyses three variables were found to be independently associated with gefitinib sensitivity and are listed in descending order of importance. The response variable is the log_10_(IC50) values of gefitinib.

After adjusting for the effects of *EGFR* and *HER2* copy numbers, the *EGFR* mutation status is significantly associated with gefitinib sensitivity as indicated in the “Estimate” column of the Table. The association coefficient is −1.5, which means after adjusting for *EGFR* and *HER2* copy number effects, the IC50 for *EGFR* wild type cell lines is 32 fold higher that that of *EGFR* mutation cell lines.

After adjusting for the effects of *EGFR* mutation and *HER2* copy numbers, the *EGFR* copy number is significantly associated with gefitinib sensitivity. The association coefficient is −0.07, which means after adjusting for *EGFR* mutation and *HER2* copy number effects, the IC50 will decrease 0.85 fold when *EGFR* relative copy number increases 1 unit.

After adjusting for the effects of *EGFR* mutation and *EGFR* copy numbers, the *HER2* copy number is significantly associated with gefitinib sensitivity. The association coefficient is −0.01, which means after adjusting for *EGFR* mutation and *EGFR* copy number effects, the IC50 will decrease 0.98 fold when *HER2* relative copy number increases 1 unit.

* to the base 10.

**Table 5:** Do KRAS mutations confer resistance to TKIs?

Mutation	n	Median of the Rank order	One-sided p value
Comparison 1			
*KRAS* mutant	12	36	0.02
Other lines excluding *EGFR* mutant lines	23	24	
Comparison 2			
*KRAS* mutant	12	21.5	0.03
Wild type for all genes	17	12	

Wilcoxon signed rank one-sided test was used to test the hypothesis that m*KRAS* confers intrinsic resistance to TKIs. We compared the median rank order of gefitinib IC50 values of m*KRAS* cell lines to all other lines excluding *EGFR* mutant lines (Comparison 1) and cell lines wild type for all the other genes tested (Comparison 2). For both analyses *EGFR* mutant cell lines were excluded as they were associated with sensitivity to TKIs.

## Discussion

In order to clarify some of the conflicting data from clinical studies, we performed an in vitro study of deregulation of the *EGFR* signaling pathway in a large panel of lung cancer cell lines and correlated the results with TKI sensitivity. In contrast to SCLC, mutations or CNGs of the studied genes were frequent in NSCLC – 84.4% of NSCLC cell lines had a mutation, one or more CNGs or both. Thus the *EGFR* pathway is deregulated at high frequency in NSCLC.

Somatic mutations or CNGs of the studied genes were rare in SCLC and further studies were limited to NSCLC. About half of the NSCLC lines had a mutation in one of the genes of the *EGFR* pathway. The relative distribution of the mutations of *EGFR* pathway genes in established NSCLC cell lines were very similar to those reported from large clinical studies [3], [8], [10], [12], [14], [43]. *EGFR* (13%) and *KRAS* (24.7%) mutations were more frequently observed than other gene mutations. We found a single mutation of *HER2* and *HER*4 and no mutations of *EGFR*3. Mutations of *BRAF* and *PIK3CA* were intermediate in frequency. In general mutations were more common in adenocarcinoma subtype and relatively rare in squamous cell carcinomas except for *PIK3CA* which was more common in squamous cell carcinomas, as also occurs in tumors [14]. Mutations were mutually exclusive except for a single cell line that had both *KRAS* and *PIK3CA* mutations. We have previously reported that mutations of *EGFR*, *KRAS*, *BRAF and HER2* were mutually exclusive in 691 resected NSCLC tumors indicating that a single activating mutation in the *EGFR*-RAS-RAF signaling pathway may be sufficient for the pathogenesis of many lung cancers [10], [14]. However, the mutational status of *PIK3CA* was not mutually exclusive, which is similar to another recent report [14]. We have postulated that the *PIK3CA* and *EGFR* signaling pathways closely interact but that PI3K signaling represents a partially independent pathway [14].

Copy number gains are believed to be an important mechanism for activation of the *EGFR* pathway genes and the downstream signaling network. CNGs have been difficult to interpret due to the differences stemming from the use of different methods. Our comparison of the three methods yielded excellent concordance for binary values after we used kappa statistics to determine the optimal cut off values for increased copy numbers. However, the three methods we compared have their individual advantages and disadvantages. CNGs result from gene over-representation due to chromosomal polysomy or focal amplification. FISH, a widely used technique to assess gene copy number takes can identify gains due to both mechanisms. On the other hand, not only is it expensive and time consuming but it analyzes only a small subset of cells (∼100 cells) to assess the copy number, and tandem segmental duplications may not always be detected. Thus intratumoral heterogeneity is difficult to assess. Both aCGH and qPCR analyze DNA from many thousands of cells, but because they compare values for the test gene against an internal standard (a reference locus), they can detect focal amplifications more readily than polysomy. In the case of qPCR, if the amplicon encompasses the reference locus, then amplification may be underestimated. In the case of aCGH, the copy number deduced from the signal ratio can appear lower than those obtained by the other two techniques, for aCGH measures relative and not absolute copy number. It does not take into account changes in ploidy and therefore will dampen ratio shifts for copy number gains in samples with a high DNA index [33]. Thus, the absolute copy number for a given chromosome region is best determined by using a combination of complementary methods [44]. Despite the shortcomings of each method, we found a high concordance among the three techniques when using binary values (normal/increased), similar to findings in a recent comparison [33]. We had most extensive data for gene copy number generated via qPCR and used it for all our further analysis.

Sixty seven percent of the NSCLC lines had CNGs for one or more *EGFR* pathway genes. CNGs for *EGFR* (40.3%), *HER2* (18.2%) and *KRAS* (14.3%) were more frequent than the other genes. *BRAF* CNGs (13%) were more common in adenocarcinoma subtype and *PIK3CA* CNGs (36.4%) were more frequent in squamous cell carcinoma as compared to the other subtypes. Other genes did not show subtype bias. Unlike mutations, CNGs were not mutually exclusive either with other CNGs or with mutations.

However, CNGs were not completely random. We identified that *EGFR* and *KRAS* CNGs were significantly more frequent in *EGFR* and *KRAS* mutant cell lines respectively. Using subcloning, we determined that in almost all mutant lines with CNGs, the mutant allele was in great excess in comparison to the wild type allele. In some lines with diploid amounts of the mutant gene, the wild type allele was absent or in minute amounts. This finding for oncogenes (as compared to tumor suppressor genes) has been described, particularly in hematologic malignancies [45], [46], and represents a form of acquired uniparental disomy. Taken together, we term this phenomenon as mutant allele specific imbalance (MASI). Thus MASI may result from specific amplification of the mutant allele, or by loss of the wild type gene, or by a combination of these events. Of interest, wild type *KRAS* may function as an inhibitor of tumorigenesis and thus represent a form of tumor suppressor gene [47]. These results suggest that the combination of two methods of activating the oncogenes *EGFR* and *KRAS* (i.e. mutation and MASI) may confer a greater growth or survival advantage to the malignant cell than a single method. These findings also indicate that mutations must precede CNGs in cells harboring both changes. Other evidence for the combination of mutations and CNGs in tumor cells exists [48], [49], [50], [51], and for the concept that mutations are the initial event [48], [49], [50], [52], [53].

*EGFR* mutations were exclusively found in adenocarcinoma histology and in never smokers or individuals with low smoke exposure (<15 PYR). In addition *EGFR* mutations were more common in a comparatively younger age group (<55 years). *EGFR* mutations did not demonstrate gender bias. Most mutant lines were derived from Caucasians, which is not surprising considering that 90% were established in the USA. In rank order of sensitivity to gefitinib, nine of the *EGFR* mutant lines were represented in the 12 most sensitive lines, in keeping with the previously discussed importance of mutations in sensitivity to TKIs.

We found an excellent concordance between the IC50 values between gefitinib and erlotinib. Depending on the previously determined in vitro threshold for gefitinib and the bimodal curve we classified our NSCLC cell lines into 3 categories: sensitive, intermediate and resistant. We had nine sensitive cell lines which included seven of the 10 *EGFR* mutant cell lines, one cell line with *EGFR* CNG and one with *HER2* CNG. Of the remaining 3 *EGFR* mutant cell lines, two had the resistance associated secondary T790M mutation. The third resistant *EGFR* mutant line had a deletion of the *PTEN* gene, a finding associated with TKI resistance in other systems [54].

Pao et al demonstrated that mutations in *KRAS* are associated with a lack of sensitivity to TKIs [28]. We tested the hypothesis that *KRAS* mutations confer intrinsic resistance to TKIs and our univariate analyses demonstrated that indeed *KRAS* mutations conferred in vitro resistance to gefitinib. Thus *KRAS* mutations are associated with both clinical and in vitro resistance to gefitinib.

None of the other gene mutations or copy number gains showed any significant correlation with gefitinib sensitivity. In the clinical setting, approximately 10–20% of NSCLC patients who do not harbor identifiable *EGFR* mutations respond partially to gefitinib. Evidently *EGFR* mutations are one of the most important but not the sole determinant of TKI response.

We evaluated the effect of *EGFR* mutation, *EGFR* CNGs and *HER2* CNGs on TKI sensitivity using a multivariate regression analysis. To summarize the multivariate analysis, all 3 parameters, i.e. *EGFR* mutations, *EGFR* CNG and *HER2* CNG, showed a correlation with the TKI sensitivity (used as a continuous variable) in decreasing order of importance. Thus three previously identified factors related to patient response to TKIs are major factors in the in vitro response. However, the most important independent factor is the presence of activating *EGFR* mutations. Our findings may assist in the prediction of response and the selection of patients for targeted therapies. Another important finding was the frequent presence of selective imbalance of the mutant form (MASI) of oncogenes in tumor cells that harbor mutations of *EGFR* and *KRAS*. While MASI may confer a selective advantage to the tumor cell, its effect on clinical course or response to therapy remains to be determined.

## Supporting Information

Table S1 (0.01 MB PDF)

Table S2 (0.01 MB PDF)

Table S3 (0.01 MB PDF)

Table S4 (0.02 MB PDF)

Table S5 (0.02 MB PDF)

Table S6 (0.01 MB PDF)

Table S7 (0.02 MB PDF)
