# Supplementary material for: Alterations in Genes of the EGFR Signaling Pathway and Their Relationship to EGFR Tyrosine Kinase Inhibitor Sensitivity in Lung Cancer Cell Lines
Source: PLoS One. 2009 Feb 24;4(2):e4576. doi: 10.1371/journal.pone.0004576 (PMC2642732; doi:10.1371/journal.pone.0004576)
Supplement: Table S1 — (0.01 MB PDF) [file pone.0004576.s001.pdf]

TABLE S1- MUTATIONS IN SCLC

				Mutation status		
Cell Line		Histology		EGFR	KRAS	PIK3CA
H	**69**	SCLC		WT	WT	WT
H	**82**	SCLC		WT	WT	WT
H	**128**	SCLC		WT	WT	WT
H	**146**	SCLC		WT	WT	WT
H	**187**	SCLC		WT	WT	WT
H	**209**	SCLC		WT	WT	WT
H	**211**	SCLC		WT	nd	WT
H	**220**	SCLC		WT	WT	WT
H	**289**	SCLC		WT	WT	WT
H	**369**	SCLC		WT	WT	nd
H	**378**	SCLC		WT	WT	WT
H	**510**	SCLC	Extrapulmonary	WT	WT	WT
H	**524**	SCLC		WT	WT	WT
H	**526**	SCLC		WT	WT	WT
H	**738**	SCLC		WT	WT	WT
H	**748**	SCLC		WT	WT	WT
H	**841**	SCLC		WT	WT	WT
H	**889**	SCLC		WT	WT	WT
H	**1045**	SCLC		WT	WT	WT
H	**1048**	SCLC	Extrapulmonary	WT	WT	**Yes**
H	**1184**	SCLC		WT	WT	WT
H	**1304**	SCLC		WT	WT	**Yes**
H	**1514**	SCLC		WT	WT	WT
H	**1607**	SCLC		WT	WT	WT
H	**1672**	SCLC		WT	WT	WT
H	**1870**	SCLC	Extrapulmonary	WT	WT	**Yes**
H	**1963**	SCLC		WT	WT	WT
H	**1994**	SCLC		WT	WT	WT
H	**2028**	SCLC		WT	WT	nd
H	**2107**	SCLC		WT	WT	WT
H	**2141**	SCLC		WT	WT	WT

H	**2171**	SCLC		WT	WT	WT
HCC	**33**	SCLC		WT	WT	WT
HCC	**954**	SCLC		WT	WT	WT
HCC	**970**	SCLC		WT	WT	WT

SCLC	Small cell lung cancer
WT	Wild type
**Yes**	Mutation
nd	Not done
