# Supplementary material for: Alterations in Genes of the EGFR Signaling Pathway and Their Relationship to EGFR Tyrosine Kinase Inhibitor Sensitivity in Lung Cancer Cell Lines
Source: PLoS One. 2009 Feb 24;4(2):e4576. doi: 10.1371/journal.pone.0004576 (PMC2642732; doi:10.1371/journal.pone.0004576)
Supplement: Table S2 — (0.01 MB PDF) [file pone.0004576.s002.pdf]

TABLE S2- GENE COPY NUMBER IN SCLC

				Gene copy number detected by qPCR						
Cell Line		Histology		EGFR	KRAS	BRAF	HER2	HER3	HER4	PIK3CA
H	**60**	SCLC		1.8	2.4	4.0	1.4	1.5	2.6	1.70
H	**128**	SCLC		2.2	1.7	1.5	1.8	1.5	2.2	1.96
H	**146**	SCLC		1.6	1.7	2.0	1.7	2.6	1.5	1.74
H	**196**	SCLC		2.0	1.1	2.0	1.6	1.5	1.8	1.90
H	**369**	SCLC		1.5	1.6	2.3	3.6	4.6	2.0	ND
H	**510**	SCLC	Extrapulmonary	1.8	1.7	1.6	2.2	2.4	3.8	3.17
H	**735**	SCLC		2.6	1.9	2.2	1.2	2.6	1.6	2.01
H	**740**	SCLC		1.5	1.7	4.4	1.5	1.8	2.4	1.93
H	**748**	SCLC		2.0	1.1	1.9	1.6	1.8	2.2	1.57
H	**774**	SCLC		1.8	1.6	4.0	3.0	1.8	1.8	ND
H	**889**	SCLC		1.6	1.7	2.7	1.6	1.8	1.2	1.56
H	**1045**	SCLC		1.6	1.7	1.9	3.2	2.2	2.8	5.30
H	**1048**	SCLC	Extrapulmonary	3.2	2.0	1.8	2.0	1.6	1.8	1.57
H	**1092**	SCLC		1.5	2.1	1.8	6.2	5.4	0.2	1.71
H	**1105**	SCLC		2.6	1.8	1.8	1.7	1.3	1.5	2.95
H	**1184**	SCLC		2.4	4.6	1.4	4.6	2	1.6	1.42
H	**1284**	SCLC		7.7	2.1	1.9	1.5	0.4	0.06	ND
H	**1304**	SCLC		3.2	1.8	2.1	2.6	2.2	1.9	3.01
H	**1417**	SCLC		1.8	1.4	2.4	1.6	1.6	1.6	3.07
H	**1450**	SCLC		4.5	1.6	1.8	1.5	1.6	2.2	2.84
H	**1514**	SCLC		1.8	2.6	2	1.8	1.5	2.4	1.59
H	**1618**	SCLC		5.1	2.5	2.3	2.2	1.5	2	2.19
H	**1870**	SCLC	Extrapulmonary	1.2	1.8	1.6	1.8	1.5	2	2.79
H	**1926**	SCLC		2	1.9	2.5	1.5	2.5	2.2	1.51
H	**1994**	SCLC		2.2	2.7	1.7	1.5	1.6	1.8	2.12
H	**2029**	SCLC		1.8	2.1	1.8	1.8	1.8	2.2	1.71
H	**2107**	SCLC		1.6	1.7	2.7	3	1.2	2	3.65
H	**2196**	SCLC		1.7	8.8	2	1.8	1.8	1.8	1.35
HCC	**954**	SCLC		1.8	1.9	2	6.6	4.7	5.3	1.56

SCLC	Small cell lung cancer
**g > 4**	Copy number gains
ND	Not done
