# Supplementary material for: Alterations in Genes of the EGFR Signaling Pathway and Their Relationship to EGFR Tyrosine Kinase Inhibitor Sensitivity in Lung Cancer Cell Lines
Source: PLoS One. 2009 Feb 24;4(2):e4576. doi: 10.1371/journal.pone.0004576 (PMC2642732; doi:10.1371/journal.pone.0004576)
Supplement: Table S3 — (0.01 MB PDF) [file pone.0004576.s003.pdf]

TABLE S3- MUTATION STATUS IN NSCLC

				Mutation status						
**Cell Line**		**Histology**	**Subtype**	**EGFR**	**KRAS**	**BRAF**	**PIK3CA**	**HER2**	**HER3**	**HER4**
Calu	**3**	NSCLC	AD	WT	WT	WT	WT	WT	WT	nd
H	**23**	NSCLC	AD	WT	Yes	WT	WT	WT	nd	nd
H	**157**	NSCLC	SQ	WT	Yes	WT	WT	WT	nd	nd
H	**226**	NSCLC	SQ	WT	WT	WT	WT	WT	WT	WT
H	**322**	NSCLC	AD	WT	WT	WT	WT	WT	WT	WT
H	**324**	NSCLC	AD	WT	WT		WT	WT	nd	nd
H	**358**	NSCLC	AD	WT	Yes	WT	WT	WT	nd	nd
H	**441**	NSCLC	AD	WT	Yes	WT	WT	WT	nd	nd
H	**460**	NSCLC	LC	WT	Yes	WT	Yes	WT	nd	nd
H	**596**	NSCLC	ADSQ	WT	WT	WT	Yes	WT	nd	nd
H	**647**	NSCLC	ADSQ	WT	Yes	WT	WT	WT	nd	nd
H	**650**	NSCLC	NS	WT	Yes	WT	WT	WT	nd	nd
H	**661**	NSCLC	LC	WT	WT	WT	WT	WT	WT	nd
H	**820**	NSCLC	AD	Yes	WT	WT	WT	WT	nd	nd
H	**838**	NSCLC	AD	WT	WT	WT	WT	WT	WT	WT
H	**920**	NSCLC	AD	WT	WT	WT	WT	WT	WT	WT
H	**1155**	NSCLC	LC	WT	Yes	WT	WT	WT	nd	nd
H	**1264**	NSCLC	ADSQ	WT	Yes	WT	WT	WT	nd	nd
H	**1299**	NSCLC	LC	WT	WT	WT	WT	WT	nd	nd
H	**1334**	NSCLC	LC	WT	WT	WT	WT	WT	WT	nd
H	**1355**	NSCLC	AD	WT	Yes	WT	WT	WT	nd	nd
H	**1395**	NSCLC	AD	WT	WT	Yes	WT	WT	nd	nd
H	**1435**	NSCLC	NS	WT	WT	WT	WT	WT	WT	nd
H	**1573**	NSCLC	AD	WT	WT	WT	WT	WT	WT	WT
H	**1648**	NSCLC	AD	WT	WT	WT	WT	WT	WT	nd
H	**1650**	NSCLC	AD	Yes	WT	WT	WT	WT	WT	nd
H	**1666**	NSCLC	AD	WT	WT	Yes	WT	WT	nd	nd
H	**1693**	NSCLC	AD	WT	WT	WT	WT	WT	WT	WT
H	**1703**	NSCLC	SQ	WT	WT	WT	WT	WT	WT	nd
H	**1755**	NSCLC	AD	WT	WT	Yes	WT	WT	WT	nd
H	**1781**	NSCLC	AD	WT	WT	WT	WT	Yes	WT	nd
H	**1792**	NSCLC	AD	WT	Yes	WT	WT	WT	nd	nd
H	**1793**	NSCLC	AD	WT	WT	WT	WT	WT	WT	WT
H	**1819**	NSCLC	AD	WT	WT	WT	WT	WT	WT	nd
H	**1975**	NSCLC	AD	Yes	WT	WT	WT	WT	WT	nd
H	**1993**	NSCLC	AD	WT	WT	WT	WT	WT	nd	nd
H	**2009**	NSCLC	AD	WT	Yes	WT	WT	WT	WT	nd
H	**2073**	NSCLC	AD	WT	WT	WT	WT	WT	WT	nd
H	**2077**	NSCLC	AD	WT	WT	WT	WT	WT	WT	nd
H	**2087**	NSCLC	AD	WT	WT	Yes	WT	WT	WT	WT
H	**2122**	NSCLC	AD	WT	Yes	WT	WT	WT	WT	nd

H	**2126**	NSCLC	LC	WT	WT	WT	WT	WT	WT	nd
H	**2170**	NSCLC	SQ	WT	WT	WT	WT	WT	nd	nd
H	**2347**	NSCLC	AD	WT	WT	WT	WT	WT	WT	nd
H	**2882**	NSCLC	NS	WT	WT	WT	WT	WT	WT	nd
H	**2887**	NSCLC	NS	WT	**Yes**	WT	WT	WT	WT	nd
H	**3255**	NSCLC	AD	**Yes**	WT	WT	WT	WT	WT	nd
HCC	**5**	NSCLC	AD	WT	WT	WT	**Yes**	WT	WT	nd
HCC	**15**	NSCLC	SQ	WT	WT	WT	WT	WT	WT	**Yes**
HCC	**44**	NSCLC	AD	WT	**Yes**	WT	WT	WT	nd	nd
HCC	**78**	NSCLC	AD	WT	WT	WT	WT	WT	WT	nd
HCC	**95**	NSCLC	SQ	WT	WT	WT	WT	WT	WT	nd
HCC	**193**	NSCLC	AD	WT	WT	WT	WT	WT	WT	WT
HCC	**364**	NSCLC	AD	WT	WT	**Yes**	WT	WT	WT	nd
HCC	**366**	NSCLC	ADSQ	WT	WT	WT	WT	WT	WT	nd
HCC	**461**	NSCLC	AD	WT	**Yes**	WT	WT	WT	WT	nd
HCC	**515**	NSCLC	AD	WT	**Yes**	WT	WT	WT	WT	nd
HCC	**827**	NSCLC	AD	**Yes**	WT	WT	WT	WT	WT	nd
HCC	**1171**	NSCLC	NS	WT	**Yes**	WT	WT	WT	WT	nd
HCC	**1195**	NSCLC	ADSQ	WT	WT	WT	WT	WT	WT	nd
HCC	**1313**	NSCLC	SQ	WT	WT	WT	WT	WT	WT	nd
HCC	**1588**	NSCLC	SQ	WT	WT	WT	WT	WT	WT	nd
HCC	**1833**	NSCLC	AD	WT	WT	WT	WT	WT	WT	nd
HCC	**1963**	NSCLC	AD	WT	WT	WT	WT	WT	WT	nd
HCC	**2108**	NSCLC	AD	WT	WT	WT	WT	WT	WT	nd
HCC	**2279**	NSCLC	AD	**Yes**	WT	WT	WT	WT	WT	nd
HCC	**2344**	NSCLC	SQ	WT	WT	WT	WT	WT	nd	nd
HCC	**2352**	NSCLC	ADSQ	WT	WT	WT	WT	WT	nd	nd
HCC	**2374**	NSCLC	LC	WT	**Yes**	WT	WT	WT	nd	nd
HCC	**2429**	NSCLC	NS	WT	WT	WT	WT	WT	WT	nd
HCC	**2450**	NSCLC	SQ	WT	WT	WT	**Yes**	WT	WT	nd
HCC	**2814**	NSCLC	SQ	WT	WT	WT	WT	WT	nd	nd
HCC	**2935**	NSCLC	NS	**Yes**	WT	WT	WT	WT	WT	nd
HCC	**3051**	NSCLC	LC	WT	WT	WT	WT	WT	WT	nd
HCC	**4006**	NSCLC	AD	**Yes**	WT	WT	WT	WT	WT	nd
HCC	**4011**	NSCLC	AD	**Yes**	WT	WT	WT	WT	nd	nd
PC	**9**	NSCLC	AD	**Yes**	WT	WT	WT	WT	nd	nd

**Yes**	Mutation
WT	Wild type
nd	Not done

AD	Adenocarcinoma
LC	Large Cell Carcinoma
SQ	Squamous Cell Carcinoma
ADSQ	Adenosquamous Carcinoma
NS	Not specific
