# Supplementary material for: Alterations in Genes of the EGFR Signaling Pathway and Their Relationship to EGFR Tyrosine Kinase Inhibitor Sensitivity in Lung Cancer Cell Lines
Source: PLoS One. 2009 Feb 24;4(2):e4576. doi: 10.1371/journal.pone.0004576 (PMC2642732; doi:10.1371/journal.pone.0004576)
Supplement: Table S4 — (0.02 MB PDF) [file pone.0004576.s004.pdf]

TABLE S4 - GENE COPY NUMBER IN NSCLC by qPCR

Cell Line		Histologic Type	Histologic subtype	COPY NUMBER USING qPCR						
				EGFR	HER2	HER3	HER4	KRAS	BRAF	PIK3CA
Calu	**3**	NSCLC	AD	4	102	2.2	2.2	2	1.8	4.87
H	**23**	NSCLC	AD	2.2	2.2	1.8	2.2	2.7	1.4	1.61
H	**157**	NSCLC	SQ	1.8	1.5	4.2	1.8	3.3	1.5	1.53
H	**226**	NSCLC	SQ	2	3	3	1.2	1.7	1.4	1.82
H	**322**	NSCLC	AD	1.6	1.8	1.6	2.2	7.6	1.9	2.38
H	**324**	NSCLC	AD	2	1.8	9.2	4.6	2	1.7	2.02
H	**358**	NSCLC	AD	3.3	1.5	1.5	1.8	7.3	2	1.61
H	**441**	NSCLC	AD	2	2.2	2.4	1.6	2.9	1.5	2.38
H	**460**	NSCLC	LC	1.6	2.2	1.5	1.8	1.3	1.9	1.94
H	**596**	NSCLC	ADSQ	5.6	3.2	2	2	1.7	1.8	1.51
H	**647**	NSCLC	ADSQ	1.6	2.2	2.4	2.2	2.2	1.9	2.33
H	**650**	NSCLC	NS	4.5	7.2	4.8	4	4.4	1.7	1.39
H	**661**	NSCLC	LC	2.6	4	2.4	1.8	4.1	2.6	1.74
H	**820**	NSCLC	AD	4	2	3	3.2	2.9	7.5	2.30
H	**838**	NSCLC	AD	2.8	2.8	2.3	2	2.8	2.2	2.87
H	**920**	NSCLC	AD	2.2	2.8	3.2	2.4	2.3	1.7	2.87
H	**1155**	NSCLC	LC	2.2	4	4.4	2.2	2.1	1.8	2.47
H	**1264**	NSCLC	ADSQ	2	2	1.5	1.6	2.6	2.2	2.53
H	**1299**	NSCLC	LC	2.4	2	6	2.2	2.1	2.1	2.45
H	**1334**	NSCLC	LC	1.5	1.5	1.6	2	2.8	3.1	2.56
H	**1355**	NSCLC	AD	2.2	2.2	5.2	3	3	1.8	1.46
H	**1395**	NSCLC	AD	2.2	1.6	1.5	1.8	1.3	1.7	1.39
H	**1435**	NSCLC	NS	4	2	1.6	1.8	1.9	1.9	1.97
H	**1573**	NSCLC	AD	11.8	1.2	2.6	2.4	2.1	2.6	1.53
H	**1648**	NSCLC	AD	4	4	1.6	1.6	2.2	1.5	2.04
H	**1650**	NSCLC	AD	4	2.2	2.4	3.2	1.7	6.2	1.42
H	**1666**	NSCLC	AD	1.8	1.5	1.5	1	1.6	1.9	1.73
H	**1693**	NSCLC	AD	4.5	8.2	1.8	1.8	1.9	2.3	3.47
H	**1703**	NSCLC	SQ	7	4.7	2.8	1.8	1.9	2.2	2.60
H	**1755**	NSCLC	AD	1.8	3.4	1.8	4.1	15.8	1.9	1.76
H	**1781**	NSCLC	AD	3	2.2	2.2	2	3.5	1.8	3.09
H	**1792**	NSCLC	AD	4.2	1.5	1.8	1.6	4	2.5	2.58
H	**1793**	NSCLC	AD	2.8	2.2	3.2	1.8	2.3	2.5	
H	**1819**	NSCLC	AD	1.8	10.2	2	1.6	1.9	1.6	5.01
H	**1975**	NSCLC	AD	2.8	2.6	2.8	2.6	1.2	1.6	1.77
H	**1993**	NSCLC	AD	3.2	1.5	1.5	2.8	2.9	1.9	2.00
H	**2009**	NSCLC	AD	1.5	2	1.5	2	11.6	1.9	2.72
H	**2073**	NSCLC	AD	10.2	2.8	2	4	2.5	1.5	2.29
H	**2077**	NSCLC	AD	2.8	4.4	1.5	2.8	1.1	2	1.86
H	**2087**	NSCLC	AD	4	2	4.3	1.6	1.8	2	1.71
H	**2122**	NSCLC	AD	2.2	1.8	1.5	1.8	1.8	2	2.31
H	**2126**	NSCLC	LC	3	2.2	2.2	1.2	1.7	2.2	1.93

H	**2170**	NSCLC	SQ	4.4	135	1.5	2.8	2.6	2.1	2.11
H	**2347**	NSCLC	AD	2	2	4.6	2.4	2	2.8	2.43
H	**2882**	NSCLC	NS	4.4	2.8	1.6	2.6	1.8	2.1	3.25
H	**2887**	NSCLC	NS	1.5	2	1.6	2.2	18.1	1	2.10
H	**3255**	NSCLC	AD	18	1.8	2.2	1.8	1.6	25	6.61
HCC	**5**	NSCLC	AD	2.2	1.6	1.6	2.4	2.7	2.1	1.52
HCC	**15**	NSCLC	SQ	2.4	1.8	1.8	2	2.2	1.7	1.66
HCC	**44**	NSCLC	AD	2.4	2.4	1.6	2	1.9	1.9	1.67
HCC	**78**	NSCLC	AD	3	1.6	1.5	3.6	2.2	1.5	1.40
HCC	**95**	NSCLC	SQ	4.1	4.2	2	2	1.9	0.6	6.41
HCC	**193**	NSCLC	AD	5.7	4	1.6	1.8	1.3	1.6	1.50
HCC	**364**	NSCLC	AD	1.6	2.2	2.8	3	2.1	1.7	1.45
HCC	**366**	NSCLC	ADSQ	4.6	2.8	2.1	3.2	3	1.4	1.31
HCC	**461**	NSCLC	AD	1.8	2.2	1.5	2.2	4.4	2.1	1.96
HCC	**515**	NSCLC	AD	1.8	1.8	4	3	2.9	2	1.61
HCC	**827**	NSCLC	AD	34	3	1.8	2.6	1.6	7.2	1.98
HCC	**1171**	NSCLC	NS	1.6	1.8	2	2.4	25.1	2.1	2.22
HCC	**1195**	NSCLC	ADSQ	1.8	2.8	2.4	2.6	3	1.4	2.68
HCC	**1313**	NSCLC	SQ	4	1.6	2.8	2	2.2	1.5	2.17
HCC	**1588**	NSCLC	SQ	4	1.4	1.8	2.4	2.8	2.2	3.38
HCC	**1833**	NSCLC	AD	2.2	1.8	2.2	2.2	3.2	2.9	1.45
HCC	**1963**	NSCLC	AD	4	2.4	1.5	2.6	72.6	2.1	2.01
HCC	**2108**	NSCLC	AD	2	4.3	2.4	2.2	2.2	1.9	2.05
HCC	**2279**	NSCLC	AD	5	2.8	2	2.4	1.6	3.2	2.21
HCC	**2344**	NSCLC	SQ	4.2	5.3	2.4	1.6	2.1	3	8.31
HCC	**2352**	NSCLC	ADSQ	2.2	2	2.6	2.4	2.4	1.8	1.94
HCC	**2374**	NSCLC	LC	2.2	2.8	2	1.5	1.9	2	2.19
HCC	**2429**	NSCLC	NS	6	2	1.5	1.6	1.7	1.8	1.67
HCC	**2450**	NSCLC	SQ	3.8	1.5	2.8	2.2	1.3	2.1	4.51
HCC	**2814**	NSCLC	SQ	4.8	1.8	1.6	1.6	1.9	2.9	10.63
HCC	**2935**	NSCLC	AD	4.4	2.4	2.2	2.8	2.2	4.5	2.10
HCC	**3051**	NSCLC	LC	1.5	2	2.6	2.4	2	2.3	1.24
HCC	**4006**	NSCLC	AD	5.2	2	2	3.2	2.1	3.6	1.73
HCC	**4011**	NSCLC	AD	8.8	2	1.5	2.4	2.4	4.4	2.68
PC	**9**	NSCLC	AD	5	2.8	1.8	2	1.7	1.5	1.82

Yes	Mutation
WT	Wild type
nd	Not done

AD	Adenocarcinoma
LC	Large Cell Carcinoma
SQ	Squamous Cell Carcinoma
ADSQ	Adenosquamous Carcinoma
NS	Not specific
