# Supplementary material for: Alterations in Genes of the EGFR Signaling Pathway and Their Relationship to EGFR Tyrosine Kinase Inhibitor Sensitivity in Lung Cancer Cell Lines
Source: PLoS One. 2009 Feb 24;4(2):e4576. doi: 10.1371/journal.pone.0004576 (PMC2642732; doi:10.1371/journal.pone.0004576)
Supplement: Table S5 — (0.02 MB PDF) [file pone.0004576.s005.pdf]

Table S5. Rank order of cell lines depending on gefitinib IC50 (n=45)

				IC50s ZD1839 (Iressa)			
**Cell Line**		**Histologic subtype**	**Mutation**	Assays	Average	SD	Rank Order
**PC**	**9**	AD	mEGFR	4	0.0309	0.0016	1
**H**	**2073**	AD	WT	4	0.0315	0.0012	2
**HCC**	**827**	AD	mEGFR	16	0.04	0.028	3
**HCC**	**2279**	AD	mEGFR	6	0.0479	0.0066	4
**H**	**3255**	AD	mEGFR	4	0.089	0.063	5
**HCC**	**2935**	AD	mEGFR	4	0.11	0.056	6
**HCC**	**4006**	AD	mEGFR	7	0.23	0.19	7
**HCC**	**4011**	AD	mEGFR	8	0.6		8
**Calu**	**3**	AD	WT	4	0.78	0.36	9
**H**	**820**	AD	mEGFR	6	3	2.2	10
**H**	**2170**	SQ	WT	4	3.2	1.6	11
**H**	**1650**	AD	mEGFR	8	11.7	4.6	12
**H**	**1573**	AD	WT	4	11.9	3.2	13
**H**	**358**	AD	mKRAS	4	12.5	1.8	14
**HCC**	**461**	AD	mKRAS	4	13.9	5.3	15
**H**	**441**	AD	mKRAS	4	15.7	1.9	16
**H**	**460**	LC	mPIK3CA	4	16.9	2	17
**H**	**1993**	AD	WT	4	17.9	7.3	18
**H**	**2087**	AD	mBRAF	3	18.4	4.9	19
**H**	**1781**	BA	mHER2	6	19	16	20
**H**	**1819**	AD	WT	4	19	2.8	21
**H**	**2882**	NS	WT	4	19.2	7.5	22
**HCC**	**193**	AD	WT	4	21.1	9.9	23
**H**	**2126**	LCC	WT	4	21.4	4.1	24
**HCC**	**95**	SQ	WT	4	24	17	25
**H**	**1975**	AD	mEGFR	4	25	8.1	26
**H**	**1299**	LC	WT	8	26.4	4.2	27
**HCC**	**1195**	MIXED	WT	4	27.6	6.7	28
**HCC**	**366**	AD	WT	9	30	31	29
**H**	**2009**	AD	mKRAS	6	33.2	8.7	30
**H**	**2122**	AD	mKRAS	4	35	16	31
**H**	**1648**	AD	WT	4	36.7	8.5	32
**HCC**	**15**	SQ	mHER4	4	52	19	33
**HCC**	**44**	AD	mKRAS	4	57.8	9.4	34
**H**	**2347**	AD	WT	4	60	43	35
**H**	**1395**	AD	mBRAF	3	71	18	36
**HCC**	**78**	AD	WT	4	81	8.4	37
**H**	**2887**	AD	mKRAS	4	110	100	38
**H**	**157**	SQ	mKRAS	4	115	18	39
**H**	**322**	AD	WT	6	120	45	40
**HCC**	**515**	AD	mKRAS	7	120	120	41
**HCC**	**1171**	AD	mKRAS	4	127	26	42
**H**	**1666**	BA	mBRAF	4	180	150	43
**H**	**1155**	LCC	mKRAS	3	183	58	44
**H**	**1355**	AD	mKRAS	4	325	24	45

m-	mutation
AD	Adenocarcinoma
LC	Large Cell Carcinoma
SQ	Squamous Cell Carcinoma
ADSQ	Adenosquamous Carcinoma
NS	Not specific
