# Supplementary material for: Alterations in Genes of the EGFR Signaling Pathway and Their Relationship to EGFR Tyrosine Kinase Inhibitor Sensitivity in Lung Cancer Cell Lines
Source: PLoS One. 2009 Feb 24;4(2):e4576. doi: 10.1371/journal.pone.0004576 (PMC2642732; doi:10.1371/journal.pone.0004576)
Supplement: Table S6 — (0.01 MB PDF) [file pone.0004576.s006.pdf]

Table S6.

Primer and probe sequences used in the real-time PCR

Gene	Location	Forward primer	Reverse primer	Probe (5' -3')
EGFR	7p11.2	ACGGCTGTCACGCCTCAC	GGCCAGGCGATGCTACTAC	TGCCGTTCAAAGCGTGACTCCTGG
β - actin	7p22.1	CTTCACCACCACGGC	CCATCTCTTGCTCGAAG	CGGGAAATCGTGCGTGACATTAAGGA
HER2	17q12	GGAGGATGTGCGGCTCG	CATGGTTGGGACTCTTGACCA	CACAGGGACTTGGCCGCTCGG
γ - actin	17q25.3	CCTGGCTGTCTTTGCAGATCA	CCACCGATCCACACCGAG	CGCACCCCCAGAGCGCAAG
HER3	12q13.2	TCTCTGGGCAGCACACAGAG	GAGTTGTGCCTGCAGTGGG	TGCCCACTCCACCCTGTACCCATC
PMCA	12q21.33	TGTTGAAGAGATTGATCACGCTG	TGTTCAGACCTCTAAACCACAAGATT	AGGGAGTTGCGGCGTGGCC
HER4	2q34	CAAGCATTGGATAATCCCGAA	TCACATACTCATCCTCGGCCT	ATCACAATGCATCCAATGGTCCACCC
NCL	2q37.1	GCAGAGATAATGACACCACATAGCA	TCCCCAGTGATTAGGAACCG	TGCTCTTCAGACCCTGTGCCCTGTC
BRAF	7q34	CGCTCTGTCGCTGATTGGTA	GCAGGTCCGACAACTGGAA	CCCCCATTCTATTCCCATCTACCGCC
ASNS	7q21.3	TGGCCAGGGCGCAGT	GGTGATAAGGCCGCAGTCTC	AGGTGTCGCGTGGTCCTGATCTAGGA

Sequences for primers for PIK3CA copy number analysis are previously stated by us (Hiromasa et. al)

Primer sequences used for gene sequencing for mutational analysis

Gene	Location	Exon / Codon	Forward primer	Reverse primer
HER3	12q13.2	18	TATGAGGAGCGGGTTGGAGTG	CACCTCTCCAGAATTCCTATGG
		19	GCATAGGATTGACCTAGGGAG	GACCCTGCTTCTACATGATTG
		20	CTGGCTGTGCACATGCTGAGTG	AATTCCTCCAGGCTTCTCTCAC
		21	ACTGCTGAGAGGTACCTTCAAG	ACCACTGGCTGCTCCCTATCC
HER4	2q34	18-20	TGCAGCTGGAGTAATTGGTG	CCGATGAACGAGTCGTCTTT
		21-23	GGCTGCCTGTTGGAGTATGT	TGGGAAGCTTCATACGATCA

Sequences for primers for other genes analysed for mutations are mentioned in our previous publications.
