# Supplementary material for: Alterations in Genes of the EGFR Signaling Pathway and Their Relationship to EGFR Tyrosine Kinase Inhibitor Sensitivity in Lung Cancer Cell Lines
Source: PLoS One. 2009 Feb 24;4(2):e4576. doi: 10.1371/journal.pone.0004576 (PMC2642732; doi:10.1371/journal.pone.0004576)
Supplement: Table S7 — (0.02 MB PDF) [file pone.0004576.s007.pdf]

**TABLE S7 - IRESSA vs TARCEVA IC50**

| | | | | IC50s Gefitinib (Iressa) | | | IC50s Erlotinib (Tarceva) | | |
|---|---|---|---|---|---|---|---|---|---|
| **Cell Line** | | **Subtype** | **Mutation** | Assays | Average | SD | Assays | Average | SD |
| **A** | **549** | AD | mKRAS | 4 | 25 | 10 | 4 | 60 | 47 |
| **Calu** | **3** | AD | WT | 4 | 0.78 | 0.36 | 4 | 1.29 | 0.77 |
| **H** | **157** | SQ | mKRAS | 4 | 115 | 18 | 4 | 128 | 19 |
| **H** | **322** | AD | WT | 6 | 120 | 45 | 5 | 56 | 53 |
| **H** | **358** | AD | mKRAS | 4 | 12.5 | 1.8 | 3 | 6.2 | 1.9 |
| **H** | **441** | AD | mKRAS | 4 | 15.7 | 1.9 | 4 | 7.1 | 1.8 |
| **H** | **460** | LC | mPIK3CA | 4 | 16.9 | 2 | 4 | 72 | 26 |
| **H** | **820** | AD | mEGFR | 6 | 3 | 2.2 | 6 | 7.1 | 4.1 |
| **H** | **1155** | LCC | mKRAS | 3 | 183 | 58 | 2 | 8.63 | |
| **H** | **1299** | LC | WT | 8 | 26.4 | 4.2 | 7 | 41.9 | 3.3 |
| **H** | **1355** | AD | mKRAS | 4 | 325 | 24 | 6 | 27 | 24 |
| **H** | **1395** | AD | mBRAF | 3 | 71 | 18 | 4 | 10.5 | 5.1 |
| **H** | **1437** | AD | WT | 3 | 62 | 12 | 4 | 12.5 | 7.2 |
| **H** | **1573** | AD | WT | 4 | 11.9 | 3.2 | ND | | |
| **H** | **1648** | AD | WT | 4 | 36.7 | 8.5 | 4 | 34 | 30 |
| **H** | **1650** | AD | mEGFR | 8 | 11.7 | 4.6 | 8 | 15 | 3.3 |
| **H** | **1666** | BA | mBRAF | 4 | 180 | 150 | 6 | 13 | 27 |
| **H** | **1770** | NE | WT | 8 | 160 | 210 | 3 | 111 | 18 |
| **H** | **1781** | BA | mHER2 | 6 | 19 | 16 | 6 | 44 | 38 |
| **H** | **1819** | AD | WT | 4 | 19 | 2.8 | 6 | 6.3 | 1.2 |
| **H** | **1975** | AD | mEGFR | 4 | 25 | 8.1 | 4 | 33 | 24 |
| **H** | **1993** | AD | WT | 4 | 17.9 | 7.3 | 4 | 5.2 | 2.8 |
| **H** | **2009** | AD | mKRAS | 6 | 33.2 | 8.7 | 4 | 25.8 | 5 |
| **H** | **2073** | AD | WT | 4 | 0.0315 | 0.0012 | 4 | 0.0313 | 0.0009 |
| **H** | **2087** | AD | mBRAF | 3 | 18.4 | 4.9 | 4 | 9.9 | 3.4 |
| **H** | **2122** | AD | mKRAS | 4 | 35 | 16 | 4 | 76.8 | 4 |
| **H** | **2126** | LCC | WT | 4 | 21.4 | 4.1 | 6 | 13 | 11 |
| **H** | **2170** | SQ | WT | 4 | 3.2 | 1.6 | ND | | |
| **H** | **2347** | AD | WT | 4 | 60 | 43 | 4 | 5.2 | 3.4 |
| **H** | **2882** | NS | WT | 4 | 19.2 | 7.5 | 4 | 66 | 18 |
| **H** | **2887** | AD | mKRAS | 4 | 110 | 100 | 4 | 101 | 7 |
| **H** | **3255** | AD | mEGFR | 4 | 0.089 | 0.063 | 3 | 0.129 | 0.016 |
| **HCC** | **15** | SQ | mHER4 | 4 | 52 | 19 | 4 | 100 | 7 |
| **HCC** | **44** | AD | mKRAS | 4 | 57.8 | 9.4 | 4 | 28 | 5.3 |
| **HCC** | **78** | AD | WT | 4 | 81 | 8.4 | 3 | 21.2 | 5.6 |
| **HCC** | **95** | SQ | WT | 4 | 24 | 17 | 4 | 18.4 | 4.6 |
| **HCC** | **193** | AD | WT | 4 | 21.1 | 9.9 | 3 | 20.5 | 4.8 |
| **HCC** | **366** | AD | WT | 9 | 30 | 31 | 4 | 11 | 2 |
| **HCC** | **461** | AD | mKRAS | 4 | 13.9 | 5.3 | 4 | 16 | 16 |
| **HCC** | **515** | AD | mKRAS | 7 | 120 | 120 | 4 | 154 | 76 |
| **HCC** | **827** | AD | mEGFR | 16 | 0.04 | 0.028 | 6 | 0.0388 | 0.0096 |
| **HCC** | **1171** | AD | mKRAS | 4 | 127 | 26 | 4 | 160 | 48 |
| **HCC** | **1195** | MIXED | WT | 4 | 27.6 | 6.7 | 3 | 175 | 28 |
| **HCC** | **1359** | LC | WT | 5 | 65 | 52 | 3 | 88 | 43 |
| **HCC** | **2279** | AD | mEGFR | 6 | 0.0479 | 0.0066 | 4 | 0.093 | 0.048 |
| **HCC** | **2935** | AD | mEGFR | 4 | 0.11 | 0.056 | 4 | 0.163 | 0.07 |
| **HCC** | **4006** | AD | mEGFR | 7 | 0.23 | 0.19 | 4 | 0.124 | 0.014 |
| **HCC** | **4011** | AD | mEGFR | 8 | 0.6 | | ND | | |
| **PC** | **9** | AD | mEGFR | 4 | 0.0309 | 0.0016 | ND | | |

| | |
|---|---|
| m- | mutation |
| ND | not done |
| AD | Adenocarcinoma |
| LC | Large Cell Carcinoma |
| SQ | Squamous Cell Carcinoma |
| ADSQ | Adenosquamous Carcinoma |
| NS | Not specific |
